# HEXploring of the HIV-1 genome allows landscaping of new potential splicing regulatory elements

**DOI:** 10.1186/1742-4690-10-S1-P78

**Published:** 2013-09-19

**Authors:** Steffen Erkelenz, Stephan Theiss, Marianne Otte, Marek Widera, Jan Otto Peter, Heiner Schaal

**Affiliations:** 1 Heinrich-Heine University Duesseldorf, Institute for Virology, Düsseldorf, Germany; 2 Heinrich-Heine University Duesseldorf, Institute for Genetics, Düsseldorf, Germany

## 

Effective selection between true and decoy splice sites is critically controlled by flanking splicing regulatory elements (SREs), which can enhance or repress splice site use. Recent experimental evidence suggests that the entire regional context of SREs rather than a single enhancer/silencer hexamer jointly contribute to splicing.

Extending the hexamer score concept [Fairbrother *et al*.: *Science* 2002, **297**:1007-13], we represent the splicing regulatory property of an entire 5’ss neighborhood by a weighted average of normalized Z-scores for all hexamers overlapping with the target region. These novel “HEXplorer” scores describe the degrees of exon- or intron-likeness (ZEI) and enhancer-likeness (ZWS) for a given region upstream of a 5’ss. These scores can be graphically represented by positive or negative oriented areas along the sequence. Mutation effects on an entire 5’ss neighborhood are then captured by comparing the HEXplorer areas of wild type and mutant sequences upstream of a 5’ss. The fundamental datasets of weak and strong 5’ss used in the definition of these HEXplorer scores were derived based on the HBond score that measures the 5’ss complementarity to U1 snRNA.

In a first test, we scanned the small non-coding HIV-1 leader exon 3 for regions enriched in SREs. Here, HEXplorer scores correctly indicated both the well-known exonic splicing silencer ESSV and the recently discovered exonic splicing enhancer ESEvpr upstream of 5’ss D3.

Next, we tested the HEXplorer’s capability to predict mutations’ potency to modify 5’ss D3 usage. We systematically examined this ESE region using various single and double mutations predicted to either alter 5’ss usage or act neutrally. In 20 tested mutations, the HEXplorer prediction correlated well with the experimentally detected level of exon inclusion.

Extending the HEXplorer approach to all HIV-1 exons, we were able to identify three novel exonic splicing enhancers that contribute to the inclusion of the viral exons 2, 2b and exon 4. All three novel ESEs were experimentally confirmed by HEXplorer predicted point-mutations. Beyond application to HIV-1 5’ss usage, the HEXplorer may also prove particularly useful as a method for assessing pathogenic human exonic mutations.

